# Importance of interaction between the matrix effect and microbial metabolism in the bioavailability of lignans

**DOI:** 10.1002/jsfa.70324

**Published:** 2025-11-18

**Authors:** Susana Langa, José Antonio Curiel, Ruiz de la Bastida, Ángela Peirotén, José María Landete

**Affiliations:** ^1^ Departamento de Tecnología de Alimentos Instituto Nacional de Investigación y Tecnología Agraria y Alimentaria (INIA‐CSIC) Madrid Spain

**Keywords:** bioaccessibility, bioavailability, enterolignans, flaxseed, health, microbiota

## Abstract

**BACKGROUND:**

Plant‐lignans are polyphenols with low bioavailability and bioactivity that are transformed by the intestinal microbiota into enterolignans. Our hypotheses are: (i) the way in which plant‐lignans are ingested in the diet affects, in a decisive way, the microbial metabolism of these compounds and (ii) an increase in lignan bioaccessibility will produce an increase in enterolignans produced by the intestinal microbiota, increasing the bioavailability of lignans ingested in the diet. Therefore, our aims were to determine how the matrix effect affects the metabolism of lignans by the intestinal microbiota and how improving the bioaccessibility of lignans ingested in the diet improves their bioavailability.

**RESULTS:**

Human faecal samples showed close to 50 times higher efficiency in the production of enterolactone (ENL) from secoisolariciresinol than from flaxseed extracts. ENL is the main enterolignan produced by the intestinal microbiota, and it is mainly produced from dihydroxy‐ENL (DHENL) via hydroxy‐ENL (HENL). Moreover, the human faecal samples were able to hydrogenate and hydroxylate the ENL. On the other hand, the fermentation of a lignan‐enriched food by *Bifidobacterium pseudocatenulatum* INIA P815 increased the concentration of lignans in the plasma and liver of mice that consumed a diet rich in lignans. DHENL, HENL and ENL showed the highest bioavailability.

**CONCLUSION:**

The matrix effect is a determining factor in the bioaccessibility and the efficiency of transformation of lignans into enterolignans by the intestinal microbiota. The reduction of the matrix effect increases the production of enterolignans (DHENL, HENL and ENL) by the intestinal microbiota, increasing the bioavailability of ingested lignans. © 2025 The Author(s). *Journal of the Science of Food and Agriculture* published by John Wiley & Sons Ltd on behalf of Society of Chemical Industry.

## INTRODUCTION

Lignans are polyphenols which are considered to be phytoestrogens.^1^ Lignans are found in a wide variety of plants, including pumpkin seeds, sesame seeds, rye, broccoli and mainly in flaxseed (*Linum usitatissimum* L.). Moreover, secoisolariciresinol diglucoside (SDG) is the most abundant lignan found in food, mainly in flaxseed.^2^, ^3^

Dietary lignans play a role in the prevention of certain diseases as well as ameliorating the symptoms of aging.^4^, ^5^, ^6^ However, plant lignans are found mainly in their glycosylated form such as SDG, pinoresinol diglucoside, matairesinol diglucoside and arctiin. Hence, they are not usually absorbed and must be metabolized by the intestinal microbiota into secoisolariciresinol (SECO), pinoresinol (PINO), matairesinol (MATA) and artigenin, respectively, prior to absorption. Later, these compounds can be transformed into enterolignans to which greater biological activity is attributed,^7^, ^8^ and, finally, target organs must absorb these compounds to exert their biological action. Enterolignans exert oestrogen agonist and antagonist effects,^9^ showing enzyme‐inhibiting properties and enhanced bioavailability and activity compared to their precursors.^10^, ^11^ Enterolignans have been associated with beneficial health effects, such as lowering the risk of acute coronary events and endometrial and breast cancer.^12^, ^13^, ^14^

Although plant lignans are found in high concentrations in some foods such as flaxseeds, the intake of these foods shows a low presence of enterolignans in plasma and target organs.^15^ The presence of enterolignans in plasma and target organs is dependent on the intake of foods rich in lignans and the microbiota.^16^, ^17^ However, other factors, such as the way in which lignans are ingested, can be important in the bioavailability of lignans. It has been shown that the relative bioavailability of enterolignans in humans is enhanced by the milling and crushing of flaxseed.^18^

**Figure 1:**
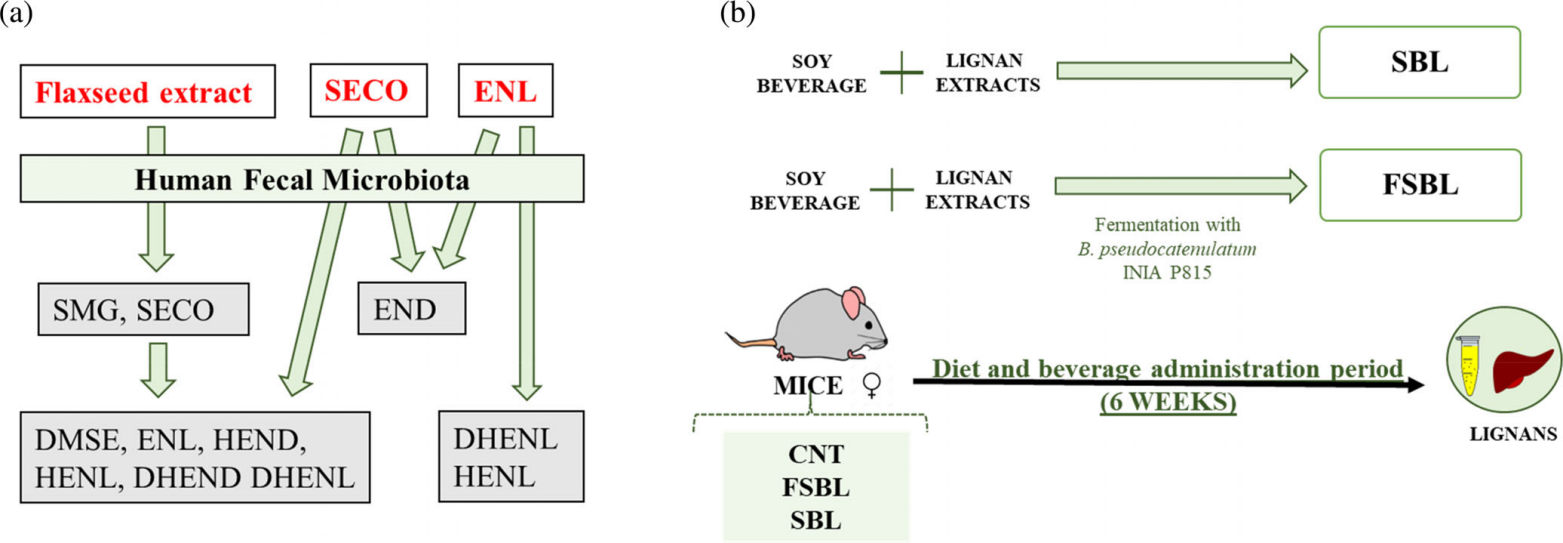
Outline of the experiments proposed in the present study. We investigated the metabolism of flaxseed extracts, SECO and ENL by human faecal samples (A) and the presence of lignans in the plasma and liver of mice that consumed a diet rich in fermented (FSBL) and non‐fermented lignans (SBL) (B). SMG, secoisolariciresinol glucoside; SECO, secoisolariciresinol; DMSE, demethylsecoisolariciresinol; END, enterodiol; ENL, enterolactone; HEND, hydroxyenterodiol; HENL, hydroxyenterolactone, DHEND. dihydroxyenterodiol; DHENL, dihydroxyenterolactone.

Our first hypothesis was that the matrix effect has a decisive influence on the way in which the intestinal microbiota metabolizes these lignans. For this reason, we studied the metabolism of flaxseed extracts, SECO and ENL, using five human faecal samples (Fig. 1).

Moreover, the bioavailability is the end result of bioaccessibility and absorption. To date, the differential absorption of lignans is unknown. However, many studies suggest that enterolignans are the lignans with the greatest bioavailability because they are found in plasma and target organs.^11^ Therefore, our second hypothesis is that, if enterolignans are the lignans that show greater bioavailability in target organs, an increase in lignan bioaccessibility will produce an increase in enterolignans produced by the intestinal microbiota, increasing the bioavailability of lignans ingested in the diet. To this end, we determined how the fermentation of foods rich in lignans affects the bioavailability of lignans in the plasma and liver of mice that consumed these foods rich in lignans (Fig. 1). Furthermore, the relative absorption of lignans could be predicted by considering bioavailability and bioaccessibility and correlating the metabolism of lignans from faecal samples with the presence of lignans in the plasma and liver of mice that consumed lignan‐enriched foods.

## MATERIALS AND METHODS

### Metabolism of lignans by the faecal samples

#### Collection of human faecal samples

Five healthy volunteers, two women and three men, aged 22–66 years, who followed an unspecified western diet, donated their faeces. They did not have a history of chronic illness nor gastrointestinal disorders. Three months prior to sample collection, they had not taken antibiotics nor probiotics. The female volunteers were not nursing or pregnant. The volunteers provided their written agreement after being fully aware of the study's objectives. Every procedure that involved human subjects complied with the 1964 Helsinki Declaration and its subsequent amendments, the institutional and/or national research committee's ethical requirements, or similar ethical criteria. The protocol was approved by the ethics committee of INIA (permission SG/RRHH‐LCH).

#### Fermentation assay of faecal samples with flaxseed extracts, SECO and ENL

Faecal suspensions were collected immediately after deposition in reduced phosphate‐buffered saline (PBS) (20 g in 100 mL of PBS) and homogenized using a Stomacher (Seward, Worthing, UK), followed by centrifugation at 200 × *g* for 1 min at 4 °C. Subsequently, 100 μL of supernatant was added to 9.9 mL of Brain Heart Infusion broth (BHI) plus 0.5 g/L of cysteine supplemented with SECO (10 mg L^–1^, 27 μm), ENL (10 mg L^–1^, 33.52 μm) and flaxseed extracts (2 g L^–1^). SECO, ENL and flaxseed extracts were individually incubated with each of the different faecal samples and incubated under anaerobic conditions (10% H_2_, 10% CO_2_ and 80% N_2_; Whitley DG250 Anaerobic workstation; Don Whitley Scientific Limited, Bingley, UK) for 24 h at 37 °C. Samples with SECO, ENL and flaxseed extracts without faecal suspensions, and samples only with faecal suspensions, were incubated in the same conditions and were used as controls. After the incubation time, the samples were frozen at −30 °C until their extraction and subsequent analysis. To validate the results of the work, at least three biological replicates were performed for all experiments.

SECO and ENL were HPLC grade and purchased from Sigma‐Aldrich (St Louis, MO, USA). LinumLifeTM EXTRA, a flaxseed extract from flax, which contains a standardized content of SDG (20%), was provided by Frutarom Netherlands BV (Veenendaal, The Netherlands).

### Effect of a diet rich in fermented and non‐fermented lignans in the presence of lignans in the plasma and liver of mice

#### Fermentation of lignan‐enriched foods by *Bifidobacterium pseudocatenulatum* INIA P815

A soy beverage (Vital; DIA, Madrid, Spain), supplemented with lignan extracts (10 g L^–1^; LinumLife EXTRA; Frutarom Netherlands BV) was autoclaved to 121 °C for 1 min. After cooling to 37 °C, this supplemented soy beverage was inoculated with *B. pseudocatenulatum* INIA P815 at an initial concentration of 6.5–7.0 log colony‐forming units mL^–1^ and incubated for 24 h at 37 °C under anaerobic conditions (10% H_2_, 10% CO_2_ and 80% N_2_; Whitley DG250 Anaerobic Workstation). The fermented soy beverage supplemented with lignan extracts (FSBL) and the non‐fermented soy beverage supplemented with lignan extracts (SBL) were stored at −30 °C until administration to the mice.

#### Animals, experimental groups and study design

Experiments were conducted by following European legislation. All study protocols were approved by the Ethical Committee on Animal Experimentation of INIA (Madrid, Spain) and were registered on the Dirección General de Agricultura y Ganaderia de la Comunidad de Madrid (Spain) (PROEX 188/17). CD1 mice (*n* = 15) were housed in an animal facility with a controlled temperature and photoperiod (14:10 h light/dark photocycle). The mice were fed either a standard diet (*n* = 18, distributed in three cages), with a basic diet low in phytoestrogens (SAFE 150; Scientific Animal Food & Engineering, Augy, Bourgogne Franche‐Comté, France). The mice fed with a standard diet were considered the control group (CNT) and did not receive any beverage. The other groups consisted of mice fed with a standard diet and a lignan‐supplemented soy beverage (SBL), and mice fed with a standard diet and a fermented version of the beverage (FSBL). The beverages were administered daily for 6 weeks in drinking bottles with 24 mL of the respective beverage, resulting in an average consumption of 4 mL per mouse per day, which is equivalent to a daily consumption of 12.5 μm SDG. The animals' body weight and food intake were controlled weekly. Blood samples were collected, from each group after 6 weeks and before euthanizing the animals following Ruiz de la Bastida *et al*.^19^ After the mice had been euthanized, the liver was dissected from each animal and weighed, then frozen in liquid nitrogen and stored at −80 °C until analysis.

#### Extraction and identification of phenolic compounds

For the fermentation assay of faecal samples, bacterial suspensions were removed by centrifuging at 5000 × *g* for 5 min. Later, phenolic compounds from 10 mL of Brain Heart Infusion (BHI) with polyphenols and the faecal suspension, as well as from the controls, were extracted twice with 2 mL of diethyl ether, and twice with 2 mL of ethyl acetate. For experiments with mice, the lignans were extracted from the plasma and liver following Ruiz de la Bastida *et al*.^19^ After extractions of lignans from the fermentation assay and plasma and liver, the solvents were evaporated at room temperature with a rotavapor, and the residue was dissolved in 300 μL of methanol/water (50:50, v/v), then filtered through a 0.22‐μm cellulose acetate filter (Millipore, Madrid, Spain), before being transferred into HPLC vials and stored at – 20 °C until analysis. Fluorescein 50 mg L^–1^ (Merck, Darmstadt, Germany), in 80% methanol was used as the internal standard. The presence of lignans shown in Table 1 was analyzed in the extracted samples by HPLC‐PAD and HPLC‐electrospray ionization/mass spectrometry (ESI/MS) using a HPLC‐pulsed amperometric detection Beckman System Gold (Beckman Coulter Inc., Fullerton, CA, USA) comprising a diode array detector. The separation of lignans was achieved on a reverse phase Nova‐Pak C18 column (300 × 3.9 mm, 4 μm) (Waters, Barcelona, Spain) with the analytical conditions described by Gaya *et al*.^20^, ^21^ Briefly, solvent A (water/acetic acid, 98:2 v/v) and solvent B (water/acetonitrile/acetic acid, 78:20:2 v/v/v) were used in a gradient at a flow rate of 1 mL min^–1^ for the first 55 min and 1.2 mL min^–1^ for the remaining time. The gradient profile was 100–20% A for 0–55 min, 20–10% A for 55–70 min, 10–5% A for 70–80 min and 100% B for 80–110 min. The detection process involved scanning between 210 and 400 nm at an acquisition speed of 1 s. A 25‐μL volume was injected. Every day, solvents A and B were made. The samples underwent double analysis. To keep the chemicals from changing over the retention period, HPLC solvents have to be created every day.

**Table 1:** Lignans detected from metabolism of flaxseed extract, SECO and enterolactone from the fecal samples, and the presence of these compounds in plasma and liver of mice after intake of soy beverage supplemented with flax extract

Lignans	Molecular formula	*m/z*	rT (min)	Flaxseed Extracts	SECO	ENL	Presence in plasma/liver
PINO	C_20_H_22_O_6_	357.134	23.4	+	−	−	−
MATA	C_20_H_22_O_6_	357.134	29.2	+	−	−	−
SMG	C_26_H_36_O_11_	523.219	15.3	+	+	−	+
SECO	C_20_H_26_O_6_	361.166	19.3	+	+	−	+
DMSE	C_19_H_24_O_6_	347.151	15.7	+	+	−	+
Demethyl‐dehydroxy‐SECO	C_19_H_24_O_5_	331.155	−	−	−	−	−
HEND	C_18_H_22_O_5_	317.140	17.9	−	−	−	−
HENL	C_18_H_18_O_5_	313.108	24.4	+	+	+	+
DHEND	C_18_H_22_O_6_	333.135	12.1	+	+	−	+
DHENL	C_18_H_18_O_6_	329.104	18.0	+	+	+	+
END	C_18_H_22_O_4_	301.144	24.2	−	+	+	+
ENL	C_18_H_18_O_4_	297.114	31.5	+	+	+	+
Enterofuran	C_18_H_20_O_3_	283.338	−	−	−	−	−

SMG, secoisolariciresinol glucoside; SECO, secoisolariciresinol; DMSE, demethylsecoisolariciresinol; END, enterodiol; ENL, enterolactone; HEND, hydroxyenterodiol; HENL, hydroxyenterolactone, DHEND. dihydroxyenterodiol; DHENL, dihydroxyenterolactone; MATA, matairesinol; PINO, pinoresinol. rT, retention time.

Moreover, mass spectra were obtained using a LC‐MS Agilent 1200 (Agilent, Palo Alto, CA, USA) chromatography system with a photodiode array detector (G1315B), thermostatted column compartment (G1316A) and a quadrupole mass spectrometer (QTOF Agilent G6530A) with an electrospray ionization interface and Masshunter Data Acquisition and Qualitative Analysis (B.40.0) as the control software. Other ESI/MS parameters were: gas flow 10 L min^–1^, range acquisition *m*/*z* 100–1000, gas temperature 350 °C, sheath gas flow 11 L min^–1^ and capillary voltage 3500 V. Moreover, the mass spectrometer operated in the negative ion mode.

Chromatographic peaks of SECO, END, ENL, MATA and PINO were identified by HPLC‐ESI/MS using the extracted ion chromatogram and confirmed by comparison of retention times with those of standards. Compounds for which standards were not available demethylsecoisolariciresinol (DMSE), dihydroxyenterodiol (DHEND), dihydroxyenterolactone (DHENL), hydroxyenterodiol (HEND) or hydroxyenterolactone (HENL) were tentatively identified according to the extracted ion chromatogram, the molecular formula and the percentage of possibility proposed by Masshunter Data Acquisition and Qualitative Analysis (B.40.0) higher than 98%. Moreover, the elution order of the lignans was similar to that shown by Quartieri *et al*.^8^ DMSE, DHEND, DHENL, HEND and HENL were quantified with calibration curves of SECO, END and/or ENL. In addition to the compounds shown in Table 1, the presence of enterofuran, MATA derivatives or those related to anhydrosecoisolariciresinol (AHS) identified by Quarteri *et al*.^8^ were also analyzed.

In addition, the presence of 3,4‐dihydroxyphenylacetic acid, 2‐(4‐hydroxyphenyl)‐propionic acid, protocatechuic acid, catechol, phloroglucinol and resorcinol were analyzed following Landete *et al* ^22^ These compounds were purchased from Sigma‐Aldrich (Sigma‐Aldrich; Merck, Darmstadt, Germany).

### Statistical analysis

Data are expressed as the mean ± SD. Differences were considered significant at *P* < 0.05. Statistical analysis of the data was performed using SPSS, version 22.0 (IBM Corp., Armonk, NY, USA). Data were analyzed by analysis of variance, using a general linear model. Comparison of means was carried out using Tukey's honestly significant difference test, as a parametric post‐hoc test that follows a significant analysis of variance.

## RESULTS

### Metabolism of flaxseed extracts by the intestinal microbiota

Microbiota from five faecal samples hydrolyzed the sugar moiety from SDG to release SMG and later SECO. SMG, as an intermediate compound of the transformation of SDG into SECO, was found in the control; however, its concentration did not show a significant variation as a consequence of the incubation of the faecal samples with the flaxseed extracts. SECO was the lignan that showed a greater increase after the incubation of the flaxseed extracts with the five faecal samples (Table 2). Controls with only faecal suspensions did not show the presence of lignans in any of the five fecal samples used in the present study.

**Table 2:** Metabolism of flaxseed extracts by faecal samples

	SMG (μm)	SECO (μm)	DMSE (μm)	DHEND (μm)	END (μm)	DHENL (μm)	HENL (μm)	ENL (μm)	MAT (μm)	PIN (μm)
Control	23.83 ± 1.32^a^	14.34 ± 0.56^a^	0.81 ± 0.16^a^	ND^a^	ND^a^	ND^a^	ND^a^	ND^a^	0.21 ± 0.02^a^	ND^a^
FS1	32.54 ± 15.70^a^	385.14 ± 67.34^c^	28.86 ± 17.98^c^	1.80 ± 0.85^b^	ND^a^	2.03 ± 1.87^b^	1.01 ± 0.34^b^	3.31 ± 0.88^c^	14.67 ± 5.76^c^	8.35 ± 4.32^b^
FS2	73.23 ± 5.87^b^	289.01 ± 23.30^bc^	6.23 ± 0.25^b^	2.25 ± 0.24^b^	ND^a^	0.63 ± 0.21^b^	NQ^a^	1.09 ± 0.75^b^	7.56 ± 1.23^b^	4.03 ± 1.45^b^
FS3	52.11 ± 9.02^ab^	245.89 ± 17.09^b^	5.05 ± 0.30^b^	3.19 ± 0.15^c^	ND^a^	0.24 ± 0.18^b^	NQ^a^	2.15 ± 1.08^bc^	6.02 ± 0.54^b^	5.53 ± 1.25^b^
FS4	43.10 ± 11.30^ab^	354.43 ± 28.55^c^	14.14 ± 1.52^b^	2.42 ± 0.35^b^	ND^a^	1.02 ± 0.45^b^	0.54 ± 0.24^b^	2.87 ± 1.55^b^	10.12 ± 1.40^c^	6.00 ± 0.67^b^
FS5	28.76 ± 12.56^a^	323.20 ± 69.00^bc^	30.78 ± 18.02^c^	1.71 ± 1.11^b^	ND^a^	2.53 ± 1.02^b^	1.24 ± 0.82^b^	3.02 ± 1.06^bc^	16.87 ± 5.67^c^	10.04 ± 3.23^bc^

^a‐c^Different lowercase letters indicate statistically significant differences (*P* < 0.05) by Tukey's test.

ND, not detected; NQ, not quantified. SMG, secoisolariciresinol glucoside; SECO, secoisolariciresinol; DMSE, demethylsecoisolariciresinol; END, enterodiol; ENL, enterolactone; HEND, hydroxyenterodiol; HENL, hydroxyenterolactone, DHEND. dihydroxyenterodiol; DHENL, dihydroxyenterolactone; MATA, matairesinol; PINO, pinoresinol.

**Figure 2:**
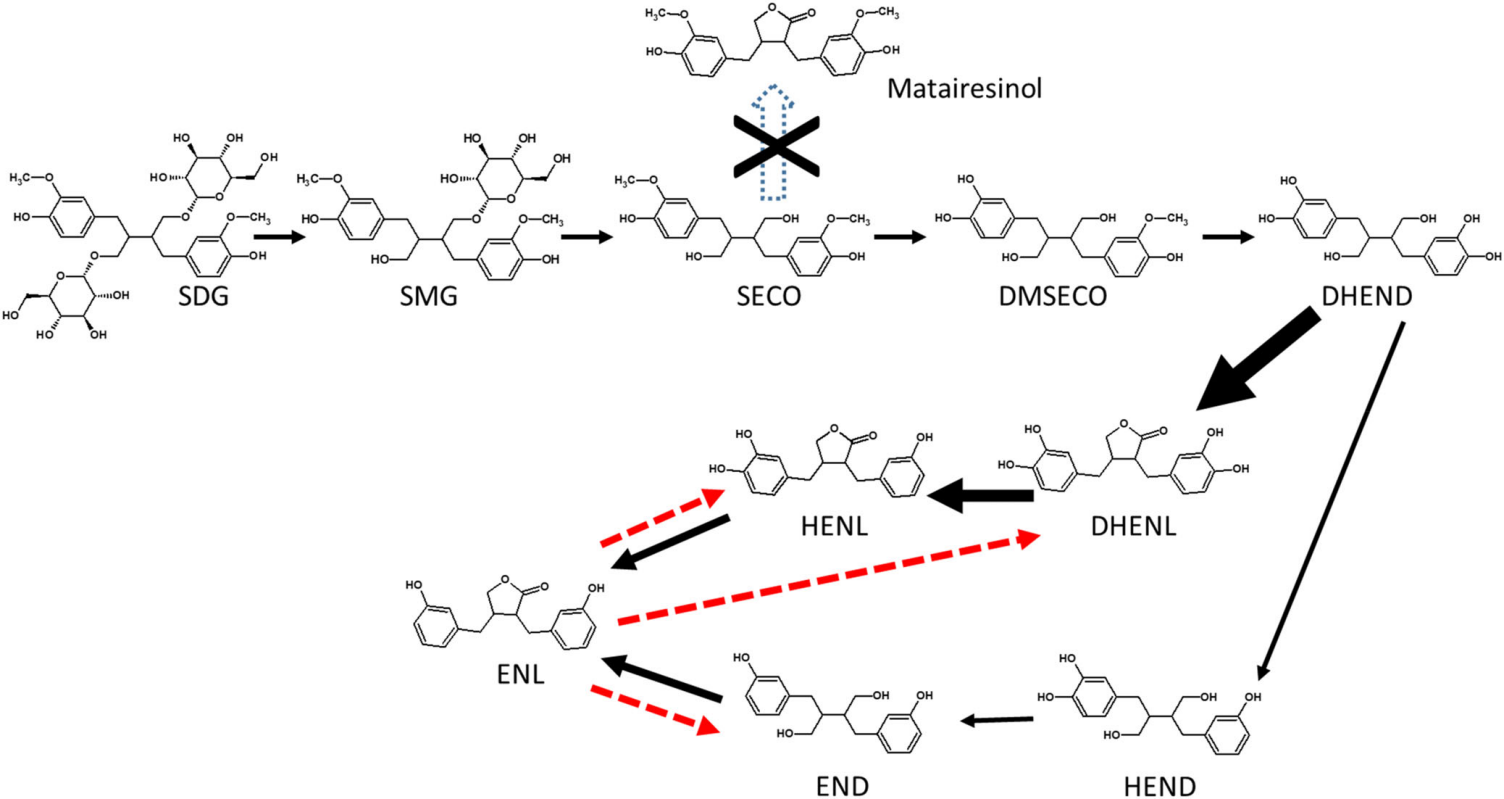
Metabolism of SDG by the intestinal microbiota. Thicker lines indicate the primary transformation pathway for that compound. Red lines indicate hydrogenation and hydroxylation reactions. SMG, secoisolariciresinol glucoside; SECO, secoisolariciresinol; DMSE, demethylsecoisolariciresinol; END, enterodiol; ENL, enterolactone; HEND, hydroxyenterodiol; HENL, hydroxyenterolactone, DHEND. dihydroxyenterodiol; DHENL, dihydroxyenterolactone;

Deglycosylation of SECO was followed by demethylation of SECO for the five faecal samples to produce DMSE and subsequently DHEND (Fig. 2). An increase in DMSE and DHEND concentrations was found in samples from all subjects (Table 2).

None of the individuals showed END production from flaxseed extracts. However, we observed that all the faecal samples produced DHENL and HENL. Subsequently, we observed the formation of ENL, confirming the production of enterolignans by the intestinal microbiota from five faecal samples.

Although low concentrations of MATA were detected in the control, and PINO was not detected, the concentration of MATA and PINO increased significantly as a consequence of the metabolism of flaxseed extracts by the five faecal samples. Moreover, neither demethyl‐dehydroxy‐SECO, hydroxy‐END, demethyl‐MATA, nor demethyl‐dehydroxy‐MATA were identified. Finally, neither AHS, nor AHS‐related compounds, such as demethyl‐AHS, didemethyl‐AHS, or didemethyl‐dehydroxy‐AHS, were identified. In addition, no enterofuran was found.

### Metabolism of SECO by the intestinal microbiota

Incubation of the intestinal microbiota with the pure compound SECO showed an important variation in the metabolism of SECO by the different faecal samples, whereas the faecal microbiota FS1 and FS5 consumed 73.3% and 52.4% of SECO, respectively, the rest of the faecal samples consumed less than 50% of the SECO after 24 h. Although an important difference in the consumption of SECO was observed, the incubation of SECO with the faecal samples showed a similar behavior in the different faecal samples. In addition to the fraction of SECO that was not metabolized, in all the faecal samples we observed the formation of DMSE, DHEND, DHENL, HENL and ENL (Table 3). END production was only observed in two of the five faecal samples (FS1 and FS5), comprising exactly the same faecal samples that showed a higher consumption of SECO. Moreover, the concentration of END produced was lower than that of the other compounds. Demethyl‐dehydroxy‐SECO and hydroxy‐END were not identified. Therefore, the metabolism of pure SECO compounds by the gut microbiota was similar to the transformation pathway from SECO derived from the metabolism of flax extract. However, two of the five individuals showed the production of END, albeit at low concentrations (Table 3).

**Table 3:** Metabolism of SECO by faecal samples

	SECO (μm)	DMSE (μm)	DHEND (μm)	END (μm)	DHENL (μm)	HENL (μm)	ENL (μm)
Control	25.45 ± 1.76^d^	0.23 ± 0.12^a^	ND^a^	ND^a^	ND^a^	ND^a^	ND^a^
FS1	7.20 ± 2.01^a^	5.01 ± 3.23^c^	1.05 ± 0.33^c^	0.44 ± 0.30^b^	3.05 ± 1.25^c^	2.95 ± 0.45^b^	6.84 ± 2.34^c^
FS2	21.78 ± 1.98^c^	2.45 ± 0.65^b^	0.40 ± 0.22^b^	ND^a^	1.05 ± 0.45^b^	NQ^a^	2.16 ± 1.08^b^
FS3	20.19 ± 0.76^c^	1.98 ± 0.35^b^	0.49 ± 0.17^b^	ND^a^	0.85 ± 0.67^b^	NQ^a^	3.98 ± 0.90^bc^
FS4	18.55 ± 2.12^b^	3.47 ± 1.05 ^cb^	0.72 ± 0.32^b^	ND^a^	0.53 ± 0.35^b^	NQ^a^	3.02 ± 1.00^bc^
FS5	12.85 ± 6.80^b^	3.30 ± 2.56^b^	1.56 ± 1.23^c^	0.52 ± 0.17^b^	3.12 ± 1.54^c^	2.88 ± 0.56^b^	4.61 ± 1.88^bc^

^a–c^Different lowercase letters indicate statistically significant differences (*P* < 0.05) by Tukey's test.

ND, not detected; NQ, not quantified. SMG, secoisolariciresinol glucoside; SECO, secoisolariciresinol; DMSE, demethylsecoisolariciresinol; END, enterodiol; ENL, enterolactone; HEND, hydroxyenterodiol; HENL, hydroxyenterolactone, DHEND. dihydroxyenterodiol; DHENL, dihydroxyenterolactone.

We did not observe the formation of MATA compound from SECO in any of the individuals (Fig. 2). Moreover, enterofuran, MATA derivatives or those related to AHS were not identified either.

### Metabolism of ENL by the intestinal microbiota

More than 90% of the initial concentration of ENL was present after 24 h of incubation with the different faecal samples. However, the appearance of END, DHENL and HENL, when the faecal samples were incubated with ENL, was noteworthy (Table 4). A concentration of less than 1% of ENL was transformed into END and HENL, and between 0.5 and 1.8% into DHENL, which appeared in all the faecal samples incubated with ENL, suggesting that the intestinal microbiota has the ability to produce the hydrogenation and the hydroxylation of ENL (Fig. 2). Even so, we can consider ENL to be a stable compound because we did not observe the production of any of the analyzed degradation compounds, such as 4‐dihydroxyphenylacetic acid, 2‐(4‐hydroxyphenyl)‐propionic acid, protocatechuic acid, catechol, phloroglucinol or resorcinol.

**Table 4:** Metabolism of ENL by faecal samples

	ENL (μm)	END (μm)	DHENL (μm)	HENL (μm)	Degradation compounds
Control	32.45 ± 0.85^a^	ND^a^	ND^a^	ND^a^	ND^a^
FS1	30.56 ± 1.95^a^	0.24 ± 0.10^b^	0.55 ± 0.20^b^	0.28 ± 0.15^a^	ND^a^
FS2	29.03 ± 0.77^a^	0.31 ± 0.18^b^	0.17 ± 0.45^b^	NQ^a^	ND^a^
FS3	30.02 ± 1.00^a^	NQ^a^	0.42 ± 0.05^b^	NQ^a^	ND^a^
FS4	29.60 ± 2.05^a^	NQ^a^	0.23 ± 0.07^b^	NQ^a^	ND^a^
FS5	30.15 ± 1.17^a^	0.15 ± 0.05^b^	0.40 ± 0.05^b^	0.12 ± 0.04^b^	ND^a^

^a‐b^Different lowercase letters indicate statistically significant differences (*P* < 0.05) by Tukey's test. ND, not detected; NQ, not quantified. END, enterodiol; ENL, enterolactone; HENL, hydroxyenterolactone; DHENL, dihydroxyenterolactone;

### Matrix effect on enterolignan production

Comparison of enterolignan production from flaxseed extracts and from SECO by the faecal samples showed a slightly higher production of enterolignans from SECO, although the difference was not significant. However, when we compared the efficiency of the production of ENL from flaxseed extracts and from SECO by the faecal samples, it could be observed that the efficiency of production from SECO is much higher than flaxseed extracts. A concentration of 2 g L^–1^ flaxseed extract contains 629 μm SDG and therefore can produce 629 μm SECO, whereas 10 mg L^–1^ SECO is 27 μm SECO. For FS1 (the faecal sample that produced a higher concentration of ENL) the transformation efficiency was 0.53% from flaxseed extracts and 25.3% from SECO, whereas, for FS2 (the faecal sample that produced a lower concentration of ENL), the transformation efficiency was 0.21% from flaxseed extracts and 8.00% from SECO. Therefore, the efficiency of transformation of SECO as pure compounds into ENL is 50 times higher for FS1 and 38 times for FS2 compared to the production of ENL from flaxseed extracts.

### Influence of fermentation of lignan‐rich food on the presence of lignans in the plasma and liver of mice

The fermentation of the lignan‐rich food by *B. pseudocatenulatum* INIA P815 produced mainly SECO (767.45 ± 35.56 μm) and lignans derived from the metabolism of SECO such as DMSE (12.25 ± 2.34 μm) and DHEND (0.95 ± 0.40 μm). Moreover, MATA (12.05 ± 3.12 μm) and PINO (3.72 ± 1.55 μm) were also produced by *B. pseudocatenulatum* INIA P815 (Table 5).

**Table 5:** Lignan content (μm) in beverages supplemented with flaxseed extract (SBL) and beverages supplemented with flaxseed extract and fermented by *B. pseudocatenulatum* INIA P815 (FSBL)

	SBL	FSBL
SMG	35.67 ± 6.25	101.34 ± 16.88
SECO	15.40 ± 4.07	767.45 ± 35.56
DMSE	NQ	12.25 ± 2.32
DHEND	NQ	0.95 ± 0.40 μm
END	ND	ND
ENL	ND	ND
MAT	1.34 ± 0.55	12.05 ± 3.12
PIN	NQ	3.72 ± 1.55

ND, not detected; NQ, not quantified. SMG, secoisolariciresinol glucoside; SECO, secoisolariciresinol; DMSE, demethylsecoisolariciresinol; END, enterodiol; ENL, enterolactone; HEND, hydroxyenterodiol; HENL, hydroxyenterolactone, DHEND. dihydroxyenterodiol; DHENL, dihydroxyenterolactone; MATA, matairesinol; PINO, pinoresinol.

An analysis of the presence of lignans in the plasma and liver of mice that had ingested soy beverages supplemented with flaxseed extracts, fermented (FSBL) and non‐fermented (SBL) by *B. pseudocatenulatum* INIA P815, demonstrated the presence of SECO, DMSE, DHEND, END, DHEN, HENL and ENL in both the plasma and liver (Table 6). These lignans have been previously identified in the metabolism of SECO by the faecal samples (Table 2). Moreover, the presence of HEND was also found in the plasma and liver (Table 6). We did not observe the presence of any glycosylated lignans, nor were enterofuran. MATA derivatives, or those related to AHS, identified either.

**Table 6:** Lignan content in mice plasma and liver that ingested fermented (FSBL) and non‐fermented (SBL) soy beverages supplemented with flaxseed extract (mice consuming a normal diet were used as a control)

		SECO	DMSE	DHEND	HEND	END	DHENL	HENL	ENL	MAT	PINO	Total Lig
Plasma	CNT	ND^a^	ND^a^	ND^a^	ND	ND^a^	ND^a^	ND^a^	ND^a^	ND	ND	ND^a^
	Non‐F	0.134 ± 0.028^b^	0.056 ± 0.014^b^	0.023 ± 0.017^b^	NQ	0.067 ± 0.010^b^	0.089 ± 0.015^b^	0.045 ± 0.012^b^	0.125 ± 0.010^b^	NQ	ND	0.539^b^
	F	0.125 ± 0.012^b^	0.072 ± 0.017^b^	0.031 ± 0.011^b^	NQ	0.101 ± 0.015^c^	0.147 ± 0.020^c^	0.043 ± 0.007^b^	0.196 ± 0.019^c^	NQ	ND	0.715^c^
	ΔBiod (%)	−0.672	28.57	34.78	—	50.75	65.17	−4.44	56.80	—	—	32.65
Liver	CNT	ND^a^	ND^a^	ND^a^	ND^a^	ND^a^	ND^a^	ND^a^	ND^a^	ND^a^	ND	ND^a^
	Non‐F	0.753 ± 0.078^b^	0.625 ± 0.044^b^	0.050 ± 0.010^b^	0.015 ± 0.011^b^	0.097 ± 0.011^b^	0.267 ± 0.163^b^	0.125 ± 0.025^b^	0.704 ± 0.156^b^	0.076 ± 0.025^b^	NQ	2.636^b^
	F	0.865 ± 0.057^b^	0.956 ± 0.102^c^	0.045 ± 0.008^b^	0.019 ± 0.005^b^	0.175 ± 0.032^c^	0.587 ± 0.098^c^	0.232 ± 0.055^c^	1.939 ± 0.225^c^	0.059 ± 0.018^b^	NQ	4.818^c^
	ΔBiod (%)	14.87	52.96	−10.00	26.67	80.41	119.85	85.60	175.43	−22.37	—	82.78

^a–c^Different lowercase letters indicate statistically significant differences (*P* < 0.05) by Tukey's test. The units were μm for plasma and μm g^–1^ for liver. ΔBiod (%): variation in the bioavailability of lignans by fermentation.

ND, not detected; NQ, not quantified. SECO, secoisolariciresinol; DMSE, Demethylsecoisolariciresinol; END, enterodiol; ENL, enterolactone; HEND, hydroxyenterodiol; HENL, hydroxyenterolactone, DHEND. dihydroxyenterodiol; DHENL, dihydroxyenterolactone; MATA, matairesinol; PINO, pinoresinol.

Although MATA and PINO were absent in either the plasma or the liver of control mice, MATA was detected in the plasma and liver of mice that had ingested FSBL and SBL, and PINO was detected only in the liver of mice that had ingested FSBL and SBL. However, the concentrations of MATA found in plasma, and of PINO in the plasma and liver, could not be quantified because they were below the limit of quantification.

The fermentation of a soy beverage supplemented with flaxseed extracts produced an increase in the presence of enterolignans in the plasma and liver of mice. Table 6 shows that the intake of FSBL produced an increase of 32.65% and 118.77% in the total lignans analyzed in plasma and liver, respectively, with respect to SBL. The most notable results were those observed in the liver. Regarding SBL, FSBL showed an increase in DHENL of 119.85%, an increase in HENDL of 85.60%, an increase in END of 80.41% and an increase in ENL of 175.43%. Furthermore, the bioavailability of the END, DHENL and ENL showed an increase of more than 50% in plasma, whereas no significant variations in the presence of SECO, DHEND and HEND were observed between mice who ingested FSBL and SBL (Table 6).

## DISCUSSION

The bioavailability of bioactive compounds is the result of bioaccessibility and absorption, and both are fundamental to the bioactivity of these compounds and their effects on human health.^23^ In the case of lignans, and other bioactive compounds such as isoflavones, it is necessary to consider the effect of the microbial metabolism on the bioavailability and the bioactivity.^24^ For this reason, the relationship of the microbial metabolism of lignans with the matrix effect, and the bioavailability of these bioactive compounds was considered in this work. The faecal samples showed between 38 and 50 times greater efficiency in the production of ENL from SECO compared to flaxseed extracts, showing that the matrix effect is a determining factor in the efficiency of transformation of lignans into enterolignans. Regarding bioaccessibility, the matrix effect of lignans has been demonstrated in different works. Kuijsten *et al*.^18^ showed that crushing and grinding flaxseed substantially improves the bioavailability of enterolignans. Heat treatments, such as roasting and frying, also affect the bioavailability of lignans.^25^ The fermentation of lignans by the fungi *Rhizopus oryzea* increases lignan release and conversion to enterolignan,^26^ and fermentation of flaxseed extracts by *B. pseudocatenulatum* INIA P815 produced an increase in the concentration of enterolignans in the plasma and liver of mice fed a fatty diet.^19^

Our first hypothesis was that the way in which lignans are found in the diet has a decisive influence on the way in which the intestinal microbiota metabolizes these lignans, affecting their bioavailability and bioactivity. The matrix effect is decisive in the metabolism of lignan and enterolignan production by the intestinal microbiota, as we demonstrate in the present study. These results agree with the idea that fermentation could be a viable strategy for increasing lignan release and conversion to enterolignan^19^, ^26^ because SECO is more bioaccessible and facilitates the action of the intestinal microbiota, as we also demonstrate in the present study.

Regarding the metabolism of lignans by the intestinal microbiota, the lignans present in the diet are mainly in glycosylated form.^27^ For this reason, the presence of aglycones, such as PINO, are not found in the controls, and MATA and SECO are found in low concentrations (Table 2). Assays from a previous study showed the formation of these compounds to be a consequence of deglycosylation reactions by the recombinant GH3 aryl‐β‐glucosidase ‘GluLm’ from *Limosilactobacillus mucosae* INIA P508,^28^ demonstrating that these compounds are mainly in glycosylated form and are transformed into PINO, MATA and SECO as a consequence of deglycosylation reactions. The glycosylated forms are metabolites with little biological activity and are not bioavailable, and so they are not found in the plasma and target organs. Therefore, the bioactivity of the lignans ingested in the diet depends on their transformation by gut bacteria in the colon.^16^, ^29^ Thus, after deglycosylation reactions, the microbiota is able to transform SECO into enterolignans through different demethylation, dehydroxylation and dehydrogenation reactions. Different studies have reported the transformation of SDG into enterolignans, with Wang *et al*.^30^ and Quartieri *et al*.^8^ having described this transformation in more depth. Both studies, as well as the present study, show the transformation of SDG into SECO through deglycosylation reactions, something widely known. Moreover, both studies, as well as the present study, demonstrate the transformation of SECO into DMSE through a demethylation reaction, and a second demethylation reaction producing DHEND. Wang *et al*.^30^ and Quartieri *et al*.^8^ also describe the transformation of DMSE into demethyl‐dehydroxy‐SECO through a dehydroxylation reaction; however, this compound was not identified in the present study. Later, from DHEND, two different reactions are possible, a dehydrogenation reaction for the production of DHENL and a dehydroxylation reaction for the production of HEND (Fig. 2). Studies by Quarteri *et al*.^8^ and Wang *et al*.,^30^ as well as the present study, demonstrate the transformation of DHEND into HEND, and HEND into END, through dehydroxylation reactions, and a subsequent dehydrogenation reaction for the formation of ENL. Wang *et al*.^30^ only described the transformation of DHEND into HEND, whereas Quarteri *et al*.^8^ and the present study show the transformation of DHEND into DHENL through a dehydrogenation reaction, and DHENL into ENL through two dehydroxylation reactions. Moreover, Quarteri *et al*.^8^ showed the production of HENL from demethyl‐dehydroxy.‐MATA, which comes from demethyl‐MATA or from demethyl‐dehydroxy‐SECO; however, neither demethyl‐dehydroxy‐MATA, nor demethyl‐dehydroxy‐SECO were identified in the present study in any of the assays performed with flaxseed extracts, SECO or in the *in vivo* assays. Regarding the identification of lignans for which there are no standard patterns, in addition to the identification according the extracted ion chromatogram, the molecular formula and the percentage of possibility proposed by Masshunter Data Acquisition and Qualitative Analysis (B.40.0) as control software, the elution order of these lignans was compared with the elution order shown by Quateri *et al*.^8^ confirming that all the lignans presented in Table 1 are well identified.

In accordance with the results observed in the present study with human faecal samples, and the presence of lignans in mouse plasma and liver, both pathways, from DHEND to HEND and subsequently END and ENL, and the transformation of DHEND to DHENL and subsequently to ENL, occur in both human and mouse faecal microbiota. However, we suggest that the pathway showing the transformation of DHEND to DHENL is a priority in relation to the results observed with flax extracts, SECO and with mice (Fig. 2). Moreover, the highest plasma ENL concentrations in the liver^19^ are consistent with the results of the metabolism of flaxseed extracts shown in the present study, and with the highest ENL concentration in faeces and urine shown by Quarteri *et al*.^8^ Other results confirming the priority transformation of ENL from DHENL and HENL are that, in the transformation of SECO into ENL by the intestinal microbiota, we observed the formation of DMSE, DHEND, END, DHENL and ENL. However, the formation of END by the intestinal microbiota was not observed in flaxseed extracts, and only two of the five faecal samples produced END from the pure compound SECO. Similarly, the production of ENL was reported in flaxseed extracts in all the faecal samples from adult individuals, whereas the production of END was found in extracts from five out of the fourteen adult subjects investigated.^21^ Moreover, the high concentration of DHENL in the liver shown in the present study is in concordance with ENL production from DHENL (Table 5).

Continuing along the pathway of enterolignan metabolism, this is the first time that the transformation of ENL into other compounds has been demonstrated, suggesting that the intestinal microbiota has the ability to produce the hydrogenation and hydroxylation of ENL (Fig. 2 and Table 4). Even so, we can consider ENL as a stable compound because we did not observe the production of any of the analyzed degradation compounds. The inability of the intestinal microbiota to degrade ENL is very important for this compound to have physiological effects even at low concentrations, unlike the degradation of equol demonstrated by Ruiz de la Bastida *et al*.^31^ using faecal samples from both equol‐producing individuals and non‐equol‐producing individuals.

In relation to other lignans, Quarteri *et al*.^8^ showed the presence of compounds derived from AHS; however, neither AHS, nor its derived compounds, were found in any of the assays developed in our work. Dehydration of SECO to AHS could be obtained chemically by acid treatment,^32^ and, to date, the gut intestinal bacteria have never been described performing this reaction.^8^ On the other hand, although MATA can be produced from SECO as a consequence of a dehydrogenation reaction, incubation of SECO with the intestinal microbiota did not show the production of MATA. Similarly, Quarteri *et al*.^8^ did not observe the formation of MATA from SECO.

Regarding the presence of lignans in FSBL and SBL, FSBL presented a high concentration of SECO, as a consequence of the deglycosylation of SDG by *B. pseudocatenulatum* INIA P815. For the first time, the production of DMSE and DHEND by a bifidobacteria strain was detected, and, although the concentrations produced of both lignans were very low. *B. pseudocatenulatum* INIA P815 has the ability to demethylate lignans.

Continuing with the consumption of FSBL and SBL by mice and the presence of lignans in plasma and liver, Ruiz de la Bastida *et al*.^19^ demonstrated how the fermentation of a soy beverage supplemented with lignan extracts increased the presence of enterolignans, primarily in the liver of mice consuming a diet rich in lignans. In the presen tstudy, we have also demonstrated that, in addition to the increase in END and ENL in the plasma and liver, the presence of other lignans such as DHENL and HENL also increased significantly in the plasma and liver. DHENL and HENL were analyzed for the first time in mouse plasma and liver in this study. Fermentation of flaxseed extract by *B. pseudocatenulatum* INIA P815 by Ruiz de la Bastida *et al*.^19^ and the present study, produced an increase in the concentration of SECO improving the bioaccessibility of SECO, and the subsequent metabolism of lignans by the intestinal microbiota produced an increase in the bioavailability of END, DHENL, HENL and ENL in the plasma and liver of mice. The results of fermentation of flaxseed extract with *B. pseudocatenulatum* INIA P815 were even more evident with the isoflavones daidzein and genistein.^19^ The fermentation of flaxseed extract by microorganisms with high glucosidase activity decreases the matrix effect by increasing the production of aglycones, which are more easily metabolized by the intestinal microbiota into more absorbable and bioavailable compounds, such as ENL. Therefore, the second hypothesis is also confirmed in the present study demonstrating that an increase in lignan bioaccessibility will produce an increase in enterolignans produced by the intestinal microbiota, increasing the bioavailability of lignans ingested in the diet. Hence, the present study, as well as that by Ruiz de la Bastida *et al*.^19^, show how the fermentation of lignan‐rich food by a bacteria with high glucosidase activity produces an increase in the bioavailability of lignans ingested in the diet.

By contrast to the results shown in both the present study and that by Ruiz de la Bastida *et al*.,^19^ Quarteri *et al*.^8^ did not show an effect of a *B. pseudocatenulatum* strain on the production of enterolignans by the intestinal microbiota. The differences observed between Quarteri *et al*.^8^ and Ruiz de la Bastida *et al*.^19^ and the present study can be attributed to the fact that Quarteri *et al*.^8^ co‐cultivated a *B. pseudocatenulatum* WC 0401 with faecal microbiota in the presence of SDG, whereas Ruiz de la Bastida *et al*.^19^ and the present study performed the fermentation of beverages enriched with flaxseed extract prior to administering the beverage to mice. Thus, although the intestinal microbiota has a high glucosidase activity for the transformation of SDG into SECO, the administration of SECO, either as pure compounds or as a result of fermentation, improves the bioaccessibility of lignans to the intestinal microbiota, facilitating the production of enterolignans in accordance with the results shown in the present study and that by Ruiz de la Bastida *et al* ^19^

The final phase related to lignan bioavailability is the absorption phase of lignans in plasma and target organs. Many studies suggest that enterolignans are the compounds with the highest bioavailability, relative to those found in plasma and target organs.^11^, ^33^ However, to date, the differential absorption of lignans is unknown. Although the microbiota of humans and mice is different, the lignans produced by human fecal samples and the lignans found in the plasma and liver of mice were similar, as shown in Tables 2, 3 and 6. These results suggest that lignan metabolism by human and mice microbiota may be slightly similar. Thus, to understand the different absorption of lignans, Table S1 in the Supporting information shows how the main lignans produced from fermentation of flaxseed extract by faecal samples FS1 and FS2 are SECO and DMSE, with SECO accounting for more than 90% of the total lignans produced by both faecal samples, whereas ENL is less than 1% of total lignans. On the other hand, Table S2 in the Supporting information shows how ENL is the lignan that appears in the highest concentration in plasma and liver, and one of the lignans that shows the highest and bioavailability together with HENL and DHENL. ENL, HENL and DHENL represent approximately 1% of the total lignans produced by the intestinal microbiota, and more than 40% of the lignans in plasma and mice (see Supporting information, Tables S1 and S2). Thus, the relative percentage of SECO produced by the faecal samples was reduced to a third in the plasma and liver of the mice that ingested SBL, while the relative percentage of DHENL, HENL, and ENL increased between 30 and 70 times in the plasma and liver of mice. In agreement with previous studies,^11^, ^33^ ENL is the most abundant lignan in plasma and liver, along with DHENL and HENL (Table 6). These three lignans showed the highest increase in the relative percentage of lignans in the plasma and liver of mice. Thus, the results show that DHENL, HENL and ENL are the most bioavailable compounds, and it can be considered that DHENL, HENL and ENL are lignans with higher absorption. Moreover, END appears in plasma and liver, and, although the concentration of END is clearly lower than the concentration of ENL, the results suggest that END must also be an enterolignan that is easily absorbed. The presence of END in the plasma and liver, and the absence of END from flaxseed extracts, could be explained by differences in the intestinal microbiota of mice and humans, and/or because it is a compound that quickly transforms into other lignans.

Therefore, the matrix effect influences the bioavailability of lignans, and, although there are differences in lignan metabolism among different fecal samples, in the present study, we have demonstrated that fermentation of lignans prior to administration to mice improves their bioavailability. Comparison between areas of the same compounds allowed us accurately determine the influence of fermentation on the bioavailability of each compound (Table 6), avoiding the problem of not having standards for some compounds. On the other hand, although the lack of certain standards for certain lignans did not allow to accurately determine the different absorption of some lignans (see Supporting information, Tables S1 and S2), we demonstrated in this work that DHENL, HENL, and ENL are lignans with higher absorption.

## CONCLUSIONS

The reduction of the matrix effect increases the production of enterolignans such as DHENL, HENL and ENL by the intestinal microbiota, increasing the bioavailability and bioactivity of lignans ingested in the diet. Hence, fermentation could be a strategy to increase lignan release and conversion to enterolignan because SECO is more bioaccessible and facilitates the action of the intestinal microbiota. Therefore, the present study demonstrates that the bioavailability of lignans is dependent on the interaction between the matrix effect and the microbial metabolism.

## CONFLICTS OF INTEREST

The authors declare that they have no conflicts of interest.

## Supporting information


**Table S1.** Percentage of SECO, DMSE, DHEND, HEND, END, DHENL, HENL and ENL with respect to the total lignans produced by the human intestinal microbiota FS1 and FS2 from lignan extracts.
**Table S2.** Percentage of SECO, DMSE, DHEND, HEND, END, DHENL, HENL and ENL with respect to the total lignans in the plasma and liver of mice that consumed a soy beverage supplemented with flaxseed extracts and unfermented.

## Data Availability

The data that support the findings of this study are available from the corresponding author upon reasonable request.

## References

[1] Patra S , Gorai S , Pal S , Ghosh K , Pradhan S and Chakrabarti S , A review on phytoestrogens: current status and future direction. Phytother Res 37:3097–3120 (2023). 37246823 10.1002/ptr.7861

[2] Rodriguez‐Leyva D , Bassett CM , McCullough R and Pierce GN , The cardiovascular effects of flaxseed and its omega‐3 fatty acid, alpha‐linolenic acid. Can J Cardiol 26:489–496 (2010). 21076723 10.1016/s0828-282x(10)70455-4 PMC2989356

[3] Touré A and Xueming X , Flaxseed lignans: source, biosynthesis, metabolism, antioxidant activity, bio‐active components, and health benefits. Compr Rev Food Sci Food Saf 9:261–269 (2010). 33467817 10.1111/j.1541-4337.2009.00105.x

[4] Seibold P , Vrieling A , Johnson TS , Buck K , Behrens S , Kaaks R *et al*., Enterolactone concentrations and prognosis after postmenopausal breast cancer: assessment of effect modification and meta‐analysis. Int J Cancer 135:923–933 (2014). 24436155 10.1002/ijc.28729

[5] Vanharanta M , Voutilainen S , Rissanen TH , Adlercreutz H and Salonen JT , Risk of cardiovascular disease–related and all‐cause death according to serum concentrations of enterolactone: Kuopio ischaemic heart disease risk factor study. Arch Intern Med 163:1099–1104 (2003). 12742810 10.1001/archinte.163.9.1099

[6] Yoder SC , Lancaster SM , Hullar MAJ and Lampe JW , Gut microbial metabolism of plant lignans: influence on human health, in Diet‐Microbe Interactions in the Gut: Effects on Human Health and Disease, ed. by Rio KTDd , Academic Press, Cambridge, Massachusetts, pp. 103–117 (2015).

[7] Landete JM , Arqués JL , Medina M , Gaya P , de la Rivas B and Muñoz R , Bioactivation of phytoestrogens: intestinal bacteria and health. Crit Rev Food Sci Nutr 56:1826–1843 (2016). 25848676 10.1080/10408398.2013.789823

[8] Quartieri A , García‐Villalba R , Amaretti A , Raimondi S , Leonardi A , Rossi M *et al*., Detection of novel metabolites of flaxseed lignans in vitro and in vivo. Mol Nutr Food Res 60:1590–1601 (2016). 26873880 10.1002/mnfr.201500773

[9] Mueller SO , Simon S , Chae K , Metzler M and Korach KS , Phytoestrogens and their human metabolites show distinct agonistic and antagonistic properties on estrogen receptor alpha (ER alpha) and ER beta in human cells. Toxicol Sci 80:14–25 (2004). 15084758 10.1093/toxsci/kfh147

[10] Kitts DD , Yuan YV , Wijewickreme AN and Thompson LU , Antioxidant activity of the flaxseed lignan secoisolariciresinol diglycoside and its mammalian lignan metabolites enterodiol and enterolactone. Mol Cell Biochem 202:91–100 (1999). 10705999 10.1023/a:1007022329660

[11] Laveriano‐Santos EP , Luque‐Corredera C , Trius‐Soler M , Lozano‐Castellón J , Dominguez‐López I , Castro‐Barquero S *et al*., Enterolignans: from natural origins to cardiometabolic significance, including chemistry, dietary sources, bioavailability, and activity. Crit Rev Food Sci Nutr 65:3764–3784 (2025). 38952149 10.1080/10408398.2024.2371939

[12] Aarestrup J , Kyrø C , Knudsen KEB , Weiderpass E , Christensen J , Kristensen M *et al*., Plasma enterolactone and incidence of endometrial cancer in a case–cohort study of Danish women. Br J Nutr 109:2269–2275 (2013). 23114205 10.1017/S0007114512004424

[13] Canivenc‐Lavier MC and Bennetau‐Pelissero C , Phytoestrogens and health effects. Nutrients 15:317 (2023). 36678189 10.3390/nu15020317 PMC9864699

[14] Buck K , Zaineddin AK , Vrieling A , Linseisen J and Chang‐Claude J , Meta‐analyses of lignans and enterolignans in relation to breast cancer risk. Am J Clin Nutr 92:141–153 (2010). 20463043 10.3945/ajcn.2009.28573

[15] Li Y , Wang F , Li J , Ivey KL , Wilkinson JE , Wang DD *et al*., Dietary lignans, plasma enterolactone levels, and metabolic risk in men: exploring the role of the gut microbiome. BMC Microbiol 22:82 (2022). 35350985 10.1186/s12866-022-02495-0 PMC8966171

[16] Senizza A , Rocchetti G , Mosele JI , Patrone V , Callegari ML , Morelli L *et al*., Lignans and gut microbiota: an interplay revealing potential health implications. Molecules 25:5709 (2020). 33287261 10.3390/molecules25235709 PMC7731202

[17] Langa S and Landete JM , Strategies to achieve significant physiological concentrations of bioactive phytoestrogens in plasma. Crit Rev Food Sci Nutr 63:2203–2215 (2023). 34470513 10.1080/10408398.2021.1971946

[18] Kuijsten A , Arts IC , van't Veer P and Hollman PC , The relative bioavailability of enterolignans in humans is enhanced by milling and crushing of flaxseed. J Nutr 135:2812–2816 (2005). 16317125 10.1093/jn/135.12.2812

[19] Ruiz de la Bastida A , Langa S , Peirotén Á , Curiel JA , Fernández‐González R , Maroto M *et al*., Fermented lignan‐enriched soy beverage ameliorates the metabolic effects of a high‐fat diet on female mice. J Agr Food Chem 3:5194–5207 (2025). 10.1021/acs.jafc.4c06947 PMC12509329 39985458

[20] Gaya P , Arqués JL , Medina M , Álvarez I and Landete JM , A new HPLC‐PAD/HPLC‐ESI‐MS method for the analysis of phytoestrogens produced by bacterial metabolism. Food Anal Methods 9:537–547 (2016).

[21] Gaya P , Medina M , Sánchez‐Jiménez A and Landete JM , Phytoestrogen metabolism by adult human gut microbiota. Molecules 21:1034 (2016). 27517891 10.3390/molecules21081034 PMC6274073

[22] Landete JM , Flavone, flavanone and flavonol metabolism from soybean and flaxseed extracts by the intestinal microbiota of adults and infants. J Sci Food Agr 102:2575–2583 (2022). 34689346 10.1002/jsfa.11599

[23] Rein MJ , Renouf M , Cruz‐Hernandez C , Actis‐Goretta L , Thakkar SK and da Silva Pinto M , Bioavailability of bioactive food compounds: a challenging journey to bioefficacy. Br J Clin Pharmacol 75:588–602 (2013). 22897361 10.1111/j.1365-2125.2012.04425.x PMC3575927

[24] Shahidi F and Peng H , Bioaccessibility and bioavailability of phenolic compounds. J Food Bioac 4:11–68 (2018).

[25] Berenshtein L , Okun Z and Shpigelman A , Stability and bioaccessibility of lignans in food products. ACS omega 9:2022–2031 (2024). 38250420 10.1021/acsomega.3c07636 PMC10795133

[26] Zaki UKH , Fryganas C , Trijsburg L , Feskens EJ and Capuano E , In vitro gastrointestinal bioaccessibility and colonic fermentation of lignans from fresh, fermented, and germinated flaxseed. Food Funct 13:10737–10747 (2022). 36178118 10.1039/d2fo02559k

[27] Landete JM , Plant and mammalian lignans: a review of source, intake, metabolism, intestinal bacteria and health. Food Res Int 46:410–424 (2012).

[28] Curiel JA , Ruiz de la Bastida a, Langa S, Peirotén Á and Landete JM, characterization and stabilization of GluLm and its application to deglycosylate dietary flavonoids and lignans. Appl Microbiol Biotechnol 108:80 (2024). 38189949 10.1007/s00253-023-12956-9 PMC10774645

[29] Baldi S , Tristán Asensi M , Pallecchi M , Sofi F , Bartolucci G and Amedei A , Interplay between lignans and gut microbiota: nutritional, functional and methodological aspects. Molecules 28:343 (2023). 36615537 10.3390/molecules28010343 PMC9822457

[30] Wang LQ , Meselhy MR , Li Y , Qin GW and Hattori M , Human intestinal bacteria capable of transforming secoisolariciresinol diglucoside to mammalian lignans, enterodiol and enterolactone. Chem Pharm Bull 48:1606–1610 (2000). 10.1248/cpb.48.1606 11086885

[31] Ruiz de la Bastida A , Langa S , Curiel JA , Peirotén Á and Landete JM , Effect of fermented soy beverage on equol production by fecal microbiota. Foods 13:2758 (2024). 39272523 10.3390/foods13172758 PMC11394804

[32] Yuan JP , Li X , Xu SP , Wang JH and Liu X , hydrolysis kinetics of secoisolariciresinol diglucoside oligomers from flaxseed. J Agric Food Chem 56:10041–10047 (2008). 18925741 10.1021/jf8020656

[33] Clavel T , Doré J and Blaut M , Bioavailability of lignans in human subjects. Nutr Res Rev 19:187–196 (2006). 19079885 10.1017/S0954422407249704

