# Supplementary material for: Importance of interaction between the matrix effect and microbial metabolism in the bioavailability of lignans
Source: J Sci Food Agric. 2025 Nov 18;106(4):2180–9. doi: 10.1002/jsfa.70324 (PMC12872247; doi:10.1002/jsfa.70324)
Supplement: Supplementary file 1 — Table S1. Percentage of SECO, DMSE, DHEND, HEND, END, DHENL, HENL and ENL with respect to the total lignans produced by the human intestinal microbiota FS1 and FS2 from lignan extracts. Table S2. Percentage of SECO, DMSE, DHEND, HEND, END, DHENL, HENL and ENL with respect to the total lignans in the plasma and liver of mice that consumed a soy beverage supplemented with flaxseed extracts and unfermented. [file JSFA-106-2180-s001.docx]

**Table 1S.** Percentage of SECO, DMSE, DHEND, HEND, END, DHENL, HENL and ENL with respect to the total lignans produced by the human intestinal microbiota FS1 and FS2 from lignan extracts.

|  | **Human microbial metabolism (%)** | |
| --- | --- | --- |
|  | **FS1** | **FS2** |
| **SECO** | 91.23 | 96.60 |
| **DMSE** | 6.83 | 2.08 |
| **DHEND** | 0.43 | 0.75 |
| **HEND** | n.d. | n.d. |
| **END** | n.d. | n.d. |
| **DHENL** | 0.48 | 0.21 |
| **HENL** | 0.24 | n.d. |
| **ENL** | 0.78 | 0.36 |
| **Total lignans** | 100 | 100 |

(n.d: no detected). SECO, secoisolariciresinol; DMSE, Demethylsecoisolariciresinol; END, enterodiol; ENL, enterolactone; HEND, hydroxyenterodiol; HENL, hydroxyenterolactone, DHEND. dihydroxyenterodiol; DHENL, dihydroxyenterolactone.

|  | **Presence of lignans in organs of mice (%)** | |
| --- | --- | --- |
|  | **Plasma** | **Liver** |
| **SECO** | 24.86 | 29.87 |
| **DMSE** | 10.39 | 24.79 |
| **DHEND** | 4.27 | 1.98 |
| **HEND** | n.d. | 0.60 |
| **END** | 12.43 | 3.85 |
| **DHENL** | 16.51 | 6.62 |
| **HENL** | 8.35 | 4.96 |
| **ENL** | 23.19 | 27.93 |
| **Total lignans** | 100 | 100 |

**Table 2S.** Percentage of SECO, DMSE, DHEND, HEND, END, DHENL, HENL and ENL with respect to the total lignans in the plasma and liver of mice that consumed a soy beverage supplemented with flaxseed extracts and unfermented.

(n.d: no detected). SECO, secoisolariciresinol; DMSE, Demethylsecoisolariciresinol; END, enterodiol; ENL, enterolactone; HEND, hydroxyenterodiol; HENL, hydroxyenterolactone, DHEND. dihydroxyenterodiol; DHENL, dihydroxyenterolactone.
